# Serum chitotriosidase-1 (CHIT1) as candidate biomarker for mitochondriopathies

**DOI:** 10.1007/s00415-025-12916-5

**Published:** 2025-02-01

**Authors:** Laura Foerster, Leila Scholle, Tobias Mayer, Ilka Schneider, Gisela Stoltenburg-Didinger, Karl-Stefan Delank, Torsten Kraya, Andreas Hahn, David Strube, Anna Katharina Koelsch, Steffen Naegel, Lorenzo Barba, Alexander E. Volk, Markus Otto, Alexander Mensch

**Affiliations:** 1 Department of Neurology, University Medicine Halle, Ernst-Grube-Str. 40, 06120 Halle (Saale), Germany; 2 Department of Neurology, St. Georg Hospital Leipzig, Leipzig, Germany; 3 Institute of Cell and Neurobiology, Charité University Medicine Berlin, Berlin, Germany; 4 Department of Orthopedic and Trauma Surgery, University Medicine Halle, Halle (Saale), Germany; 5 Department of Pediatric Neurology, Justus-Liebig-University Gießen, Gießen, Germany; 6 Department of Neurology, Alfried Krupp Krankenhaus Rüttenscheid, Essen, Germany; 7 Institute for Human Genetics, University Hospital Hamburg-Eppendorf, Hamburg, Germany

**Keywords:** CHIT, Chitotriosidase, Mitochondriopathies, Diagnostic biomarker, FGF21, GDF15

## Abstract

**Background:**

Neuromuscular diseases (NMDs) and mitochondriopathies are rare and heterogeneous disorders. Diagnosis is often difficult and delayed, partly due to the lack of reliable biomarkers. Chitotriosidase (CHIT1) as a candidate marker for lysosomal storage diseases is elevated in Niemann pick disease type C as a prototype of this group of diseases. Most recently, a relevant role of the lysosomal pathway in mitochondriopathies has been discussed, but markers of lysosomal involvement have not been investigated. Therefore, the aim of this study was to evaluate CHIT1 concentrations in a broad spectrum of NMDs and mitochondriopathies.

**Methods:**

CHIT1 serum concentration of 151 patients with NMD or primary mitochondriopathy was determined by enzyme-linked immunosorbent assay, and compared to 38 healthy controls and 8 patients with Niemann pick disease type C. Results were controlled for age, sex, CRP and CHIT1 polymorphism, and compared to several established markers (CK, FGF21, GDF15).

**Results:**

CHIT1 levels were not altered in NMDs, but significantly increased in mitochondriopathies, within the range of Niemann-Pick patients. Compared to the established biomarkers, CHIT1 and FGF21 showed a similar diagnostic performance, while better results were found for GDF15. However, there was a tendency for higher CHIT1 concentrations in patients with central nervous system involvement (MELAS syndrome), while FGF21 and GDF15 were not relevantly altered in these patients. Consequently, a combination of biomarkers including CHIT1 provided the best overall diagnostic performance.

**Conclusions:**

Serum CHIT1 concentration is significantly elevated in mitochondriopathies compared to healthy controls and other NMD, identifying CHIT1 as potential complementary biomarker in mitochondriopathies.

**Supplementary Information:**

The online version contains supplementary material available at 10.1007/s00415-025-12916-5.

## Introduction

Neuromuscular disorders (NMDs) and mitochondriopathies include a wide range of rare diseases. Although heterogeneous in clinical presentation and pathophysiology, NMDs share muscular weakness as a predominant feature. While muscle weakness is by definition the central phenotypic feature in NMDs, mitochondriopathies—although often showing relevant muscle involvement—can present with a variety of organ manifestations, not necessarily including muscle. Due to their rarity, heterogeneity and overlapping symptoms, diagnosis in both NMDs and mitochondriopathies can be challenging. As a result, clinicians and patients often face a significant delay in diagnosis. For example, Lagler et al*.* report a median time from symptom onset to diagnosis of 144 months for the late-onset variant of Pompe disease [1]. In contrast, there is an increasing number of causal treatments for individual NMDs, including Pompe disease and spinal muscular atrophy [2, 3]. Hence, there is an urgent need for biomarkers that can facilitate the diagnostic process and serve as surrogates for disease progression.

For NMDs, only a few biomarkers have been established for individual entities (e.g. neurofilaments for amyotrophic lateral sclerosis or HEX4 for Pompe disease), while a suitable biomarker is lacking for the majority of diseases [4, 5]. In mitochondriopathies, serum levels of the fibroblast growth factor 21 (FGF21) and growth differentiation factor 15 (GDF15) have been suggested as mitochondrial biomarker candidates. However, FGF 21 and GDF15 levels have been shown to be influenced by several conditions. These include cardiovascular diseases, malignancy, age, cardiometabolic risk factors (diabetes, hypertension, smoking, low HDL), antiinflammatory drugs (NSAIDs) and nutritional challenges such as fasting conditions or ketogenic diet [6–8]. Thus, there is an imminent need for additional biomarkers for both NMDs in general and mitochondriopathies in particular to facilitate the diagnostic pathway and allow disease monitoring. Recently a relevant role of the lysosomal pathway in primary and secondary mitochondriopathies has been discussed, while markers of lysosomal involvement have not been investigated so far [9–13].

Human chitotriosidase (CHIT1) is considered to be such a marker of lysosomal involvement, since elevated levels have been demonstrated in several lysosomal storage diseases, including Gaucher disease, GM1-gangliosidosis and Niemann-Pick disease type A/B/C [14–17]. In addition, some studies suggest that CHIT1 may also be elevated in lysosomal diseases with primary muscle involvement, including Pompe disease [16, 18].

CHIT1 is a chitinase secreted by phagocytes [19]. It has also been identified in the lysosomes of macrophages [20]. The enzyme shows activity against chitin-containing pathogens in vitro and in vivo, suggesting a relevant role in the human immune response [19]. However, total enzyme deficiency can be compensated without significant deterioration [21]. There are several polymorphisms of the CHIT1 gene that lead to CHIT1 deficiency, the most common being a 24 bp duplication in exon 10 [21, 22]. The presence of this duplication in a homozygous state leads to a complete loss of enzyme activity, while the residual abundance and activity in heterozygotes is controversial [22–24]. It is important to consider this polymorphism in clinical evaluation [23].

The aim of the present work was to investigate the ability of CHIT1 to serve as a potential blood-based biomarker in NMD and mitochondriopathies to facilitate the diagnosis and management of these rare disease entities.

## Methods

### Patients and control subjects

In the present study, serum samples from a total of 197 subjects were analysed, including 38 healthy subjects (mean age: 50.66 ± 19.58 years; m: 18, f: 20), 151 patients with neuromuscular disorders (NMD) and 8 patients with Niemann Pick type C (mean age: 19.00 ± 11.77 years; m: 5, f: 3) as a benchmark for elevated CHIT1 levels. The patients with NMD were divided into three groups: hereditary (n = 90; mean age: 54.29 ± 14.00 years; m: 38, f: 52) and inflammatory (n = 27; mean age: 64.81 ± 10 18; m: 14, f: 13) myopathies as well as mitochondriopathies (n = 34; mean age: 53.18 ± 18.80 years; m: 9, f: 26). Mitochondriopathies were further sub-grouped according to the clinical diagnosis, including chronic progressive external ophthalmoplegia (CPEO, n = 17), CPEO-plus syndromes (n = 6), mitochondrial encephalomyopathy with lactic acidosis and stroke-like episodes (MELAS, n = 7), Leber hereditary optic neuropathy (LHON, n = 3), and one patient with ataxia (detailed clinical and genetic information available in Suppl. Tab. 1. All diagnoses were pre-confirmed in clinical routine using the diagnostic standard for each disease, including genetic testing for hereditary myopathies and mitochondriopathies, and application of the relevant clinicohistoserological criteria for inflammatory myopathies. The 24-bp duplication polymorphism in *CHIT1* was tested if DNA-samples were available (n = 81). Detailed clinical and demographic characteristics of the study populations can be found in Table 1.

**Table 1:** Clinical and demographic characteristics of the studied cohort

Disease	n	Mean age (y)	Standard deviation	Sex	
				m	f
**HER**	**90**	**54.29**	**14.00**	**38**	**52**
LOPD	21	52.00	14.57	8	13
MD I	6	44.33	16.27	5	1
MD II	22	60.64	9.87	3	19
FSHD	12	47.75	14.62	8	4
OPMD	8	60.75	6.16	5	3
MATR3	10	54.10	14.76	5	5
ANO5	4	59.50	7.05	3	1
McArd	7	50.86	20.32	1	6
**INF**	**27**	**64.81**	**10.18**	**14**	**13**
IBM	9	64.67	8.52	8	1
NKM	7	62.86	8.40	4	3
PM-Mito	3	72.33	12.86	0	3
OLM	8	63.88	12.87	2	6
**MITO**	**34**	**53.18**	**18.80**	**9**	**26**
CPEO	17	58.94	13.26	4	13
CPEO+	6	57.33	18.29	1	5
LHON	3	31.33	9.74	1	2
MELAS	7	40.71	18.75	3	4
ataxia	1	83	0	0	1
**NPC**	**8**	**19.00**	**11.77**	**5**	**3**
**CTR**	**38**	**50.66**	**19.58**	**18**	**20**

The main categories are highlighted in bold, the individual diseases in normal font

*HER* hereditary myopathies, *LOPD* late-onset Pompe’s disease, *MD I* myotonic dystrophy type I, *MD II* myotonic dystrophy type II, *FSHD* fazioscapulohumeral muscular dystrophy, *OPMD* Okulopharyngeal muscular dystrophy, *MATR3* MTR3-associated myopathy, *ANO5* Limb-girdle muscular dystrophy R12, *McArd* Morbus McArdle, *NPC* Niemann Pick type C, *INF* inflammatory myopathies, *IBM* inclusion body myositis, *NKM* necrotizing myopathy, *PM-Mito* polymyositis with mitochondrial pathology, *OLM* overlapmyositis, *MITO* mitochondriopathies, *CPEO* chronic progressive external ophthalmoplegia, *LHON* Leber hereditary optic neuropathy, *MELAS* mitochondrial encephalomyopathy with lactic acidosis and stroke-like episodes

### Blood samples collection and biomarker analysis

Serum samples were collected according to standard procedures and centrifuged at 4000 rpm for 10 min at 4 °C. Aliquots were stored at − 80 °C until testing. Serum CHIT1 concentrations were determined using the CircuLex Human Chitotriosidase ELISA Kit (MBL Life Science, USA) according to the manufacturer's instructions. Samples were measured in duplicate.

Routine diagnostics were used to determine creatine kinase and C-reactive protein levels. Fibroblast growth factor 21 (FGF21) and growth differentiation factor 15 (GDF15) were measured using the ELLA microfluidic system (Bio-Techne, Minneapolis, USA). Samples were analysed in triplicate. For all analyses, the intra- and inter-assay coefficients of variability were calculated to be < 10% and < 20%, respectively.

To assess the presence of the 24-bp duplication polymorphism in *CHIT1* (exon 10), DNA was isolated from EDTA blood using the Qiagen EZ1&2 DNA Blood Kit and analysed as described before [23].

### Statistical analysis

Statistical analysis was performed using GraphPad Prism, version 8.3.0 (Graphpad software, Boston, USA). Differences between the groups were calculated using Kruskal–Wallis test (followed by Dunn-Bonferroni post-hoc test) or Mann–Whitney test for continuous variables, as applicable. For categorical variables, Chi-Squared Test was applied. Spearman’s correlation coefficient was used to assess potential correlations between the studied parameters. Linear and logistic regression models were built to test associations between biomarker concentrations and clinical variables, including adjustment for age and sex. Variables that were tested significant at univariate analysis were included in multivariable models. Moreover, to test the diagnostic accuracy of individual biomarkers, receiver operating characteristic (ROC) analyses were performed. For biomarker combinations, area under the curve (AUC) values were derived from multivariable generalised linear models (GLMs). Best cutoffs were found by maximising the Youden’s index. Statistical significance was set at p < 0.05.

## Results

### Influencing factors on serum CHIT1 concentrations

#### Determination of the frequency of CHIT1 polymorphism

**Fig. 1:**
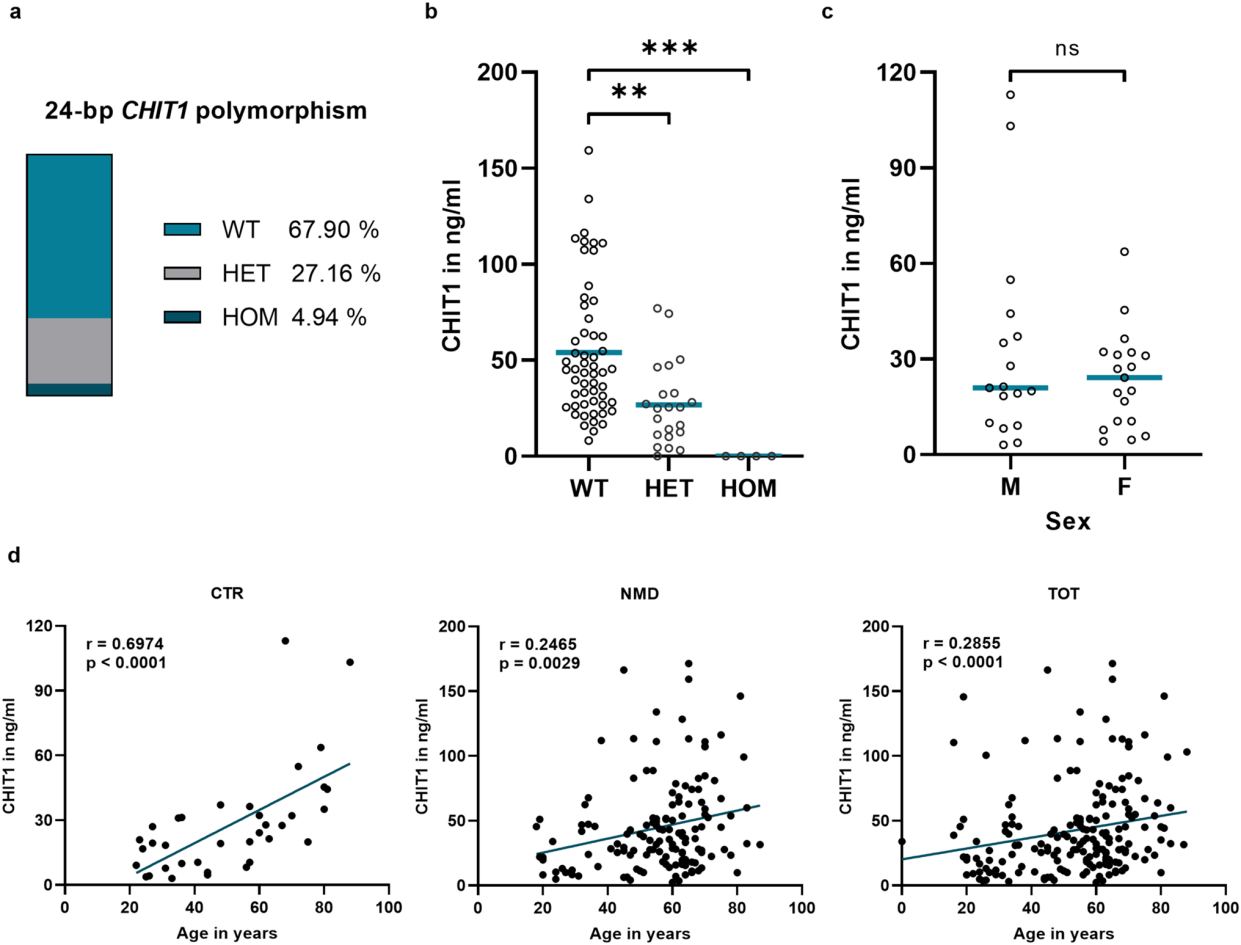
Potential factors influencing serum CHIT1 concentration. **a** Occurrence of the 24-bp duplication polymorphism in *CHIT1* with representation of the relative distribution. **b** Mean and individual values of CHIT1 concentration in heterozygous (HET) and homozygous (HOM) carriers of the polymorphism, as well as homozygous carriers of the wild type allele (WT). **c** CHIT1 concentrations in healthy males (M) and females (F). Line at median. **d** Spearman correlation of CHIT1 and age in healthy controls (CTR), patients with neuromuscular disorders (NMD) including hereditary, inflammatory and mitochondrial pathologies and total cohort (TOT). r = correlation factor

Of the 197 patients included in this study, DNA was available in 81 cases to determine the frequency of the 24-bp duplication polymorphism in *CHIT1*. Among the subjects, 4.94% carried the polymorphism in a homozygous state, 27.16% were heterozygous, and 67.90% carried wild-type alleles (Fig. 1a). Comparing the chitotriosidase (CHIT1) serum concentrations between the groups, homozygous duplication carriers consistently showed concentrations below the measurable range. The concentration of CHIT1 was significantly lower in the heterozygous allele carriers (26.70 ng/ml ± 21.30 ng/ml, p = 0.0017) compared to wild-type (54.06 ng/ml ± 34.77 ng/ml) (Fig. 1b). Based on these results, individuals with CHIT1 concentrations below the measurable range (n = 10; 5.08% of all studied specimen) were considered homozygous carriers of the 24-bp duplication polymorphism, even without genetic testing. These individuals (including those with genetic confirmation of homozygous state of the polymorphism) were excluded from further statistical analysis to avoid a relevant bias. Individuals who were found to be carriers of the heterozygous allele were subsequently enclosed for further analyses. Detailed information on the frequency of the polymorphism in the different subgroups can be found in Suppl. Tab. 2.

#### Epidemiological factors—age and sex

Among control samples (CTR), there was no significant difference in CHIT1 concentration between male and female subjects (Fig. 1c). However, there was a strong positive correlation between CHIT1 and age (r = 0.6974, p < 0.0001). Also, in all patients with neuromuscular disorders (NMD) including hereditary, inflammatory, and mitochondrial pathologies as well as the total cohort, a weak association could be observed (Fig. 1d). Among the group of inflammatory myopathies (INF; 64.81 ± 10.18 years), a significantly higher mean age was detected compared to CTR (50.66 ± 19.58 years; p = 0.0037) as well as hereditary myopathies (HER; 54.29 ± 14.00 years; p = 0.0085). There was no significant difference between all other groups. All groups were sex matched.

#### Paraclinical parameters—CK and CRP

No significant correlation was found between CHIT1 and creatine kinase in a nonparametric analysis within CTR. Although in CTR the C-reactive protein (CRP) did not reveal a significant correlation with CHIT1, a weak positive association in NMD (r = 0.2199, p = 0.0083) and the total cohort (r = 0.2224, p = 0.0023) could be observed. A simple linear regression indicated an association between CHIT1 and CRP (β = 0.67 [95%-CI: 0.13–1.20], p = 0.0148), age (β = 0.65 [95%-CI: 0.36–0.94], p < 0.0001) as well as the presence of a NMD (β = 17.07 [95%-CI: 4.75–29, 0.38], p = 0.0069). The subsequent multiple linear regression confirmed this assumption (Suppl. Tab. 3).

### CHIT1 concentrations in different neuromuscular diseases

**Fig. 2:**
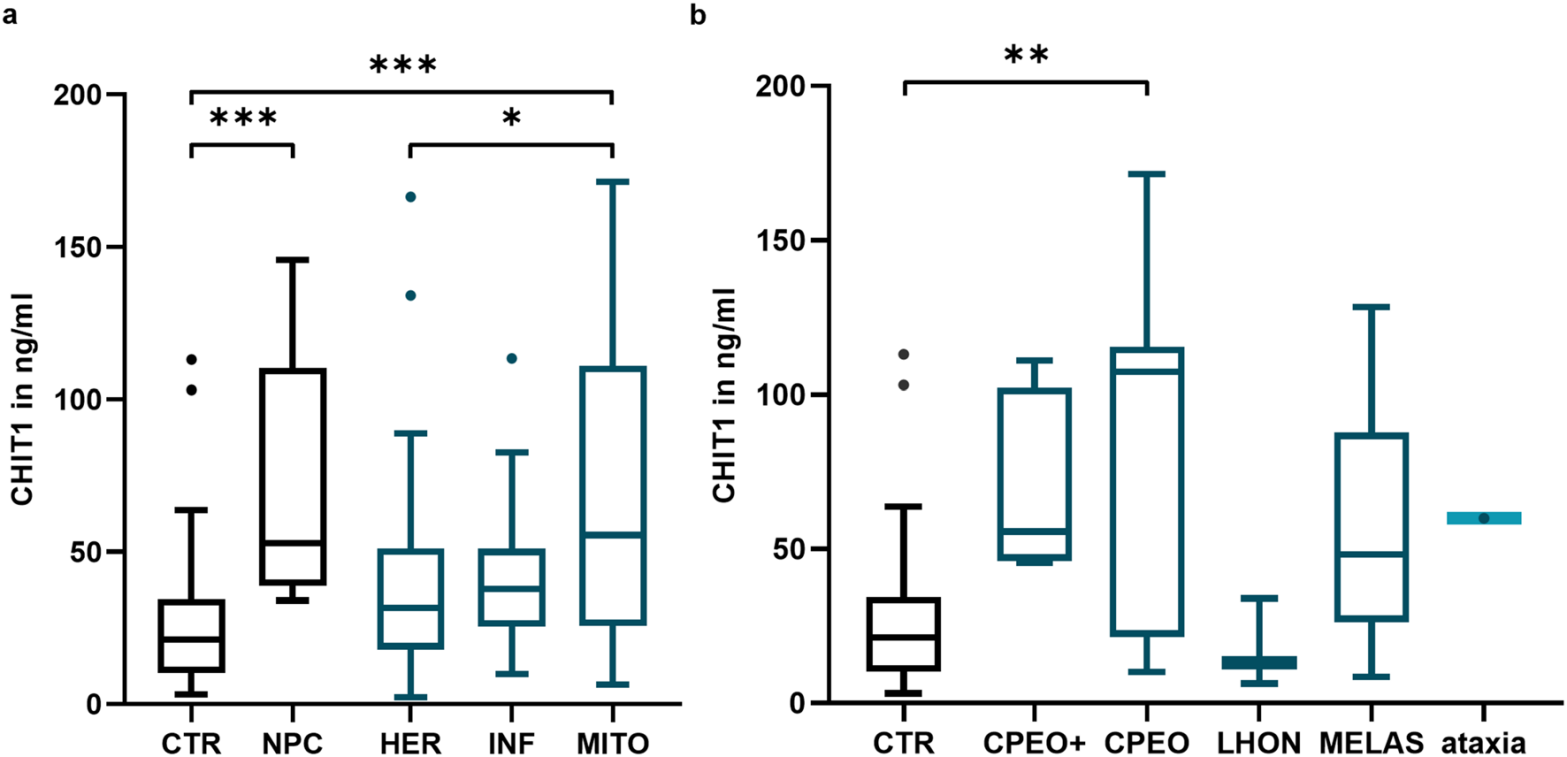
CHIT1 concentrations in patients with neuromuscular diseases. **a** CHIT1 concentrations in hereditary myopathies (HER), inflammatory myopathies (INF) and mitochondriopathies (MITO), compared to healthy controls (CTR) and Niemann Pick disease type C (NPC) as diseased control with expected elevated CHIT1 concentration. **b** Comparison of CHIT1 concentrations in different groups of mitochondriopathies (CPEO: chronic progressive external ophthalmoplegia; LHON: Leber hereditary optic neuropathy; MELAS: mitochondrial encephalomyopathy with lactic acidosis and stroke-like episodes) and CTR. Level of significance is indicated as follows: p < 0.05*, p < 0.01**, p < 0.001***, p < 0.0001****

Figure 2a summarises the serum CHIT1 concentrations in the NMD subgroups studied, while Suppl. Tab. 4 shows detailed results. The mean CHIT1 concentration in CTR was 27.77 ng/ml (± 24.62 ng/ml). In patients with NPC, a significantly increased CHIT1 concentration was observed (74.55 ng/ml ± 43.97 ng/ml, p = 0.0005). In NMD, CHIT1 levels in HER (37.11 ± 27.84 ng/ml, p = 0.2425) and INF (41.65 ± 25.77 ng/ml, p = 0.0913) were not significantly altered compared to CTR. Interestingly, this also applied for the 21 LOPD patients studied (Suppl. Figure 1). However, patients with mitochondriopathies (MITO) showed significantly elevated CHIT1 serum concentrations (68.32 ± 48.42 ng/ml) compared to both CTR (p = 0.0001) and HER (p = 0.0163). Notably, CHIT1 levels in MITO patients tended to be in the same range as those in Niemann Pick type C samples. Regarding specific subgroups of mitochondriopathies, CHIT1 levels were significantly higher in patients with CPEO (82.51 ± 56.39 ng/ml, p = 0.0077). There was also a trend towards higher CHIT1 concentrations in CPEO-plus (68.87 ± 29.14 ng/ml, p = 0.0628) and MELAS (56.59 ± 41.29 ng/ml, p = 0.8090) samples, although this was not significant, most likely due to the small group size. Patients with LHON (17.76 ± 14.45 ng/mL, p ≥ 0.9999) did not show alterations in CHIT1 levels (Fig. 2b, Suppl. Tab. 5).

Simple logistic regression performed for CTR vs. MITO including CHIT1, CRP, age and sex as independent variables confirmed CHIT1 as an independent prognostic factor for the presence of a mitochondriopathy (Table 2, p = 0.0007).

**Table 2:** Influence of several variables on the ability to discriminate between CTR vs. MITO

Variables		CTR vs. MITO				
		β	95% CI	OR	95% CI	p-value
CHIT1		0.03	0.015 to 0.051	1.031	1.015 to 1.052	0.0007
CRP		0.251	0.015 to 0.667	1.286	1.015 to 1.949	0.1585
Age		0.007	− 0.017 to 0.032	1.007	0.983 to 1.032	0.5748
Sex		0.916	− 0.058 to 1.942	2.5	0.944 to 6.974	0.0705

*CTR* Healthy controls, *MITO* mitochondriopathies, *CHIT1* chitotriosidase 1, *CRP* C-reactive protein, *OR* odds ratio, *95%* *CI* 95% confidence interval

### Diagnostic accuracy of CHIT1 compared to mitochondrial biomarkers FGF21 and GDF15

**Fig. 3:**
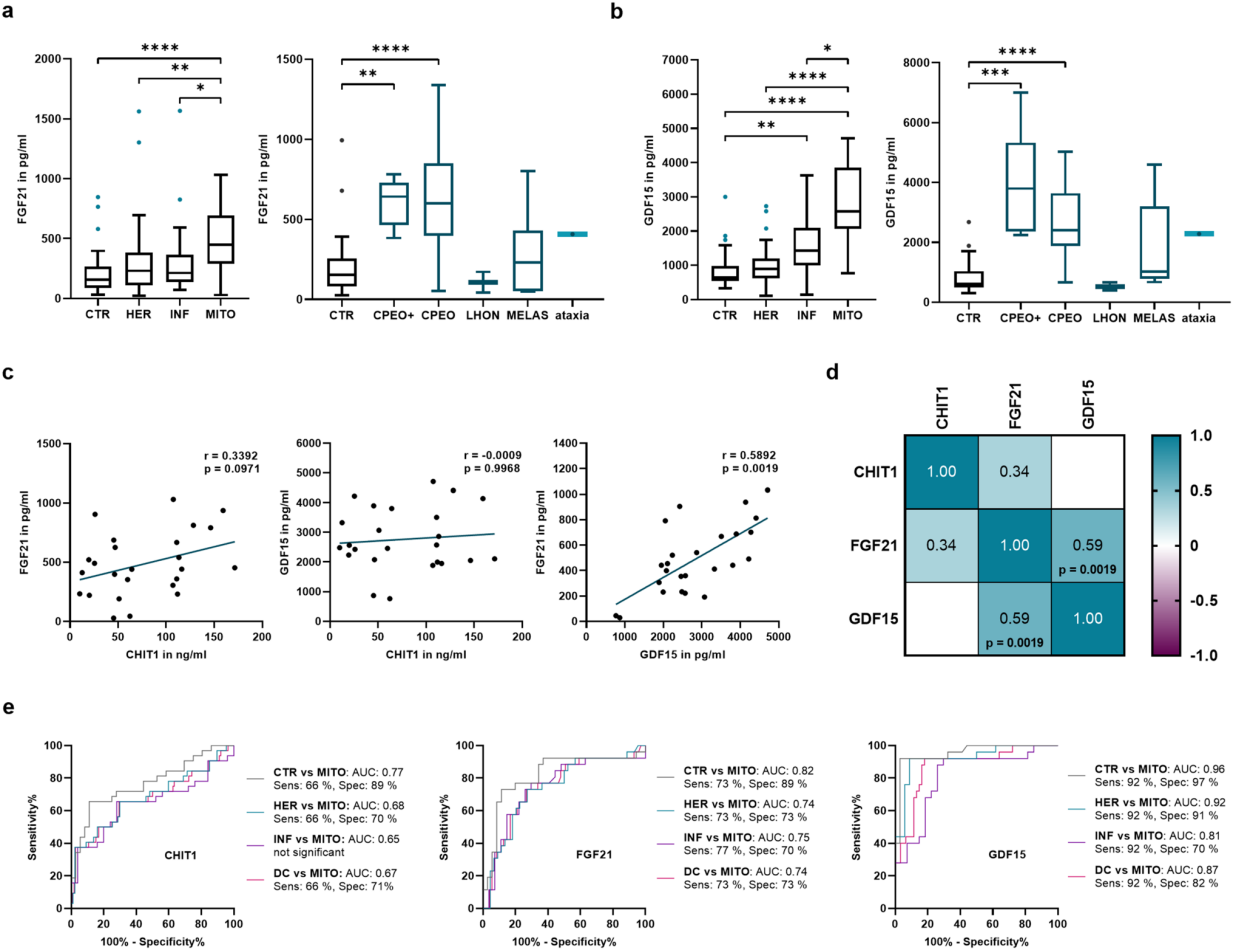
Diagnostic performance of CHIT1 in comparison to mitochondrial biomarkers FGF21 and GDF15. **a**, **b** Comparison of FGF21 (a) and GDF15 (b) concentrations between groups of NMD and CTR (left) as well as comparison between the subgroups of MITO and CTR (right) **c:** Individual correlation of CHIT1, FGF21 and GDF15. **d** Correlation matrix of CHIT1, FGF21 and GDF15. For significant correlations, p-value is indicated in the respective field. **e** ROC analyses of CHIT1, FGF21 and GDF15 comparing CTR, HER, INF and DC against MITO. AUC, Sensitivity and Specificity are given in the legend for each respective analysis. Level of significance is indicated as follows: p < 0.05*, p < 0.01**, p < 0.001***, p < 0.0001****. *CHIT1* chitotriosidase, *FGF21* fibroblast growth factor 21, *GDF15* growth differentiation factor 15, *NMD* neuromuscular disorders, *CTR* healthy controls, *HER* hereditary myopathies, *INF* inflammatory myopathies, *MITO* mitochondriopathies, *CPEO* chronic progressive external ophthalmoplegia, *LHON* Leber hereditary optic neuropathy, *MELAS* mitochondrial encephalomyopathy with lactic acidosis and stroke-like episodes, *DC* diseased controls (including HER and INF), *ROC* receiver operating characteristics, *AUC* area under the curve, *Sens* sensitivity, *Spec* specificity

To test the performance of CHIT1 in comparison to the established mitochondrial biomarkers fibroblast growth factor 21 (FGF21) and growth differentiation factor 15 (GDF15), both biomarkers were measured in the cohort studied. Compared to CTR (211.90 ± 188.80 pg/ml; p < 0.0001), serum concentrations of FGF21 were significantly higher in MITO (493.50 ± 268.10 pg/ml), while not altered in HER and INF (Fig. 3a). GDF15 levels were significantly elevated in MITO (2828.00 ± 1075.00 pg/ml; p < 0.0001) and INF (1584.00 ± 905.70 pg/ml; p = 0.0014) compared to CTR (844.40 ± 539.20 pg/ml), while they were not elevated in HER (Fig. 3b). Regarding the MITO subgroups, significantly elevated FGF21 and GDF15 levels were observed in subgroups with primary muscular phenotype (CPEO, CPEO-plus), while not significantly altered in all other subgroups (Fig. 3a, b). Detailed results and statistical information are provided in *Suppl. Tab. 4 and 5*.

Among MITO, CHIT1 did not correlate with either FGF21 or GDF15. However, there was a positive correlation between FGF21 and GDF15 (r = 0.5892, p = 0.0019) (Fig. 3c, d).

Receiver operating characteristic analysis was performed to compare the ability of CHIT1, FGF21 and GDF15 to discriminate MITO from CTR, diseased controls (DC; including HER and INF), and HER as well as INF separately (Fig. 3e). Irrespective of the group tested, the AUC as well as sensitivity (sens.) and specificity (spec.) were most favourable for GDF15 (MITO vs. CTR: AUC 0.96, sens. 0.92, spec. 0.97; MITO vs. DC: AUC 0.87, sens. 0.92, spec. 0.82). There was no substantial difference between the results observed for CHIT1 (MITO vs. CTR: AUC 0.77, sens. 0.66, spec. 0.89; MITO vs. DC: AUC 0.67, sens. 0.66, spec. 0.71) and FGF21 (MITO vs. CTR: AUC 0.82, sens. 0.73, spec. 0.89; MITO vs. DC: AUC 0.74, sens. 0.73, spec. 0.73). Detailed information regarding the ROC analyses of CHIT1, FGF21 and GDF15 can be found in *Suppl. Tab. 6*. Using generalised linear models to test the diagnostic performance of different biomarker combinations, the combination of all three biomarkers resulted in the highest AUC (MITO vs. CTR: 0.97 [0.929–0.999]; MITO vs. DC: 0.92 [0.861–0.981]), though not significant (Suppl. Tab. 7).

### Clinical applicability of CHIT1, FGF21 and GDF15 as diagnostic biomarkers for mitochondriopathies

To transfer the results into a clinical application, the individual cut-offs derived from ROC analyses of CHIT1, FGF21 and GDF15 were used to create dichotomous categories (positive or negative for the respective biomarker). Using this approach, patients with mitochondriopathy were identified with a sensitivity of 100% (95%-CI: 86.20–100.0%) and a comparably satisfying specificity (81.25% [95%-CI: 64.69–91.11%) when one of the three biomarkers was positive (Table 3). With increasing stringency (two out of three or all three biomarkers positive), specificity increased significantly while sensitivity decreased. When only CHIT1 and GDF15 were used, sensitivity was again 100% (95%-CI 86.20–100.0%), while specificity increased to 87.50% (95%-CI (71.93–95.03%) (one out of two positive). In this respect, the combination of CHIT1 and GDF15 outperformed all other combinations, including the currently used combination of GDF15 and FGF21.

**Table 3:** Approach to the clinical applicability of different biomarker combinations

	PPV	NPV	Sens (%)	95% CI (%)	Spec (%)	95% CI (%)	Likelihood ratio	
C/F/G ≥ 1 +	0.80	0.81	100.0	86.20–100.0	81.25	64.69–91.11	5.33	
C/F/G ≥ 2 +	0.95	0.84	83.33	64.15–93.32	96.88	84.26–99.84	26.67	
C/F/G all 3 +	1.00	0.84	54.17	35.07–72.11	100.0	89.28–100.0	n.d	
C/F ≥ 1 +	0.79	0.93	91.67	74.15–98.52	81.25	64.69–91.11	4.89	
C/F all 2 +	1.00	0.84	54.17	35.07–72.11	100.0	89.28–100.0	n.d	
C/G ≥ 1 +	0.86	1.00	100.0	86.20–100.0	87.50	71.93–95.03	8.00	
C/G all 2 +	1.00	0.84	66.67	46.71–82.03	100.0	89.28–100.0	n.d	
F/G ≥ 1 +	0.88	0.94	91.67	74.15–98.52	90.63	75.78–96.76	9.78	
F/G all 2 +	0.94	0.84	70.83	50.83–85.09	96.88	84.26–99.84	22.67	

*PPV* positive predictive value, *NPV* negative predictive value, *Sens* sensitivity, *Spec* specificity, *95% CI* 95% confidence interval, *C* chitotriosidase 1, *F* fibroblast growth factor 21, *G* growth differentiation factor 15

Tsygankova et al. Ref. [39]

## Discussion

There is an urgent need for biomarkers to facilitate the diagnosis and clinical monitoring of NMD and mitochondriopathies, given the delay in diagnosis on the one hand and the emerging therapeutic options on the other. In this regard, the present study is the first to investigate the potential of CHIT1 as a biomarker in a larger NMD and mitochondriopathy cohort, focusing on different disease entities.

Interestingly, a significant increase in serum CHIT1 levels was observed in mitochondriopathies compared to unaffected controls, whereas no relevant changes were observed in hereditary and inflammatory myopathies (Fig. 2a). To the best of our knowledge, this is the first study to demonstrate an increase in CHIT1 in mitochondrial disorders. Samples from patients with Niemann-Pick diseases type C were used as a biomarker benchmark to establish CHIT1 levels in relation to a prototype of a lysosomal storage disease [16, 17]. CHIT1 levels in patients with mitochondriopathies were found to be elevated within the range of NPC patients. This further emphasises the relevance of the observed CHIT1 changes. A possible confounding effect of factors previously suggested to influence CHIT1 levels was carefully excluded (Fig. 1, Table 2, Suppl. Tab. 3) [25–28]. Interestingly, no relevant changes of CHIT1 concentrations were observed in patients with late-onset Pompe disease, a lysosomal storage disorder with primary muscle involvement (Suppl. Figure 1). However, all patients studied were receiving enzyme replacement therapy (ERT). In other lysosomal storage diseases, serum CHIT1 concentrations decreased significantly after ERT initiation [15]. A similar mechanism may be considered in Pompe disease. Therefore, further studies including treatment-naive patients are mandatory to evaluate the role of CHIT1 in Pompe disease.

The exact mechanisms leading to elevated CHIT1 serum concentrations in mitochondriopathies remain unknown. Lysosomes have been implicated in maintaining mitochondrial integrity through the mechanism of mitophagy [29, 30]. Consistently, increased lysosomal activity has been implicated in several primary mitochondrial disorders where there is a relevant fraction of defective mitochondria [9–12]. CHIT1 may therefore reflect the involvement of lysosomal activation in mitochondriopathies. In ALS, the elevation of CHIT1 levels in cerebrospinal fluid appears to originate from a subpopulation of microglial cells [5, 31]. This increase could not be detected in serum, or only to a lesser extent, suggesting that the small increase stems from the CNS [5, 32]. However, in lysosomal storage disorders, macrophages are considered the primary source of serum CHIT1 [33]. Although mitochondrial diseases are quite heterogeneous in terms of clinical presentation and pathophysiology, recent studies suggest that immune dysfunction is a potent driver of disease progression [34]. Some reports suggest a relevant deregulation of the adaptive immune system in specific disease entities, which may require activation of components of the innate immune response (including macrophages) [35, 36]. However, the underlying mechanisms are poorly understood, and macrophage activation has not been systematically addressed in mitochondriopathies.

To assess the value of CHIT1 as a novel biomarker for mitochondriopathies, a comparison with the established mitochondrial biomarkers FGF21 and GDF15 was performed (Fig. 3a, b). While the results of the ROC analysis to discriminate patients with mitochondriopathies from healthy controls were basically comparable for CHIT1 and FGF21, GDF15 showed better discrimination than both FGF21 and CHIT1 (Fig. 3e, Suppl. Tab. 6). Similar results were gained with respect to the differentiation of mitochondriopathies and other neuromuscular disorders (disease controls). The data regarding the diagnostic performance of FGF21 and GDF15 are in line with previous results, further ensuring the validity of the results obtained in this study [37]. Thus, the results of the present study may favour GDF15 over FGF21 and CHIT1 in terms of its ability to identify patients with mitochondriopathy. However, there are multiple confounding factors that affect both FGF21 and GDF15 levels. Therefore, independent biomarkers appear essential to further improve patient identification and monitoring. There was a strong correlation between serum levels of FGF21 and GDF15, whereas CHIT1 did not correlate with either FGF21 or GDF15 (Fig. 3c, d). Therefore, CHIT1 may represent a novel biomarker that is independent of FGF21 and GDF15 and may be particularly useful in conditions where these biomarkers are altered due to other underlying diseases. In this study, FGF21 and GDF15 were predominantly elevated in patients with a primary muscular phenotype (CPEO, CPEO-plus), while not significantly altered in mitochondriopathies with concomitant CNS manifestation (MELAS). In these patients CHIT1 levels tended to be higher, though not statistically significant, most likely due to the small group size.

Consequently, in a generalised linear model, the combination of CHIT1, FGF21 and GDF15 discriminated better between mitochondriopathies and other neuromuscular diseases than the established combination of FGF21 and GDF15 alone (Suppl. Tab. 7). The clinical applicability of these statistical models is further supported by dichotomising the biomarker results of the individual patients (positive or negative for the individual biomarker) (Table 3). This approach increased the diagnostic ability to a virtually perfect sensitivity (100%) when at least one of the three biomarkers (CHIT1, FGF21 or GDF15) was positive, while the specificity was still considerably high (81%). Interestingly, a similar sensitivity (100%) was obtained when only CHIT1 and GDF15 were used, while specificity increased (87%). This may further support a combination of biomarkers reflecting different aspects of the pathophysiology of mitochondriopathies. By analogy with the amyloid-tau-neurodegeneration (A/T/N) classification in neurodegenerative diseases, a similar approach could be considered for mitochondriopathies, including biomarkers for metabolic disturbance (FGF21), apoptosis/cellular stress (GDF15) and lysosomal dysfunction (CHIT1) [38]. This may better reflect the clinical and pathophysiological complexity and heterogeneity of mitochondriopathies.

Interestingly, patients with Leber hereditary optic neuropathy (LHON) did not show relevant alterations in the biomarkers studied. In accordance with this study, previous reports have shown FGF-21 and GDF-15 to be within normal ranges in LHON-patients [39]. To date, there is no evidenced explanation for this finding. It could be speculated that the very localised pathology affecting only the optic nerve may not result in systemic alterations of the respective biomarkers.

This study has some obvious limitations. The size of the cohort studied seems generally adequate given the rarity of the condition. However, individual subgroups are comparatively small and unevenly sized, which may complicate interpretation of the data. Nevertheless, the observed changes appear to be robust in terms of statistical significance. However, multicentre evaluations in larger cohorts are mandatory. This will further ensure the validity of the observed changes. Although basic clinical data were included in the present study, detailed phenotypic information was lacking. Especially with regard to the clinically heterogeneous group of mitochondriopathies, in-depth phenotyping could contribute to a better understanding of the results obtained and should be considered for further studies dealing with biomarkers in mitochondrial disorders. In addition, the use of CHIT1 as a biomarker is limited by the frequent occurrence of genetic polymorphisms in the *CHIT1* gene that can lead to partial or complete loss of enzyme function [22]. As EDTA and DNA samples were not available for all patients in this study, the most common polymorphism could only be determined for 81 patients (Fig. 1a). In our cohort, the 24-bp duplication polymorphism was detected in 4.94% of the samples in a homozygous state, which is similar to the literature [23]. Our data show a significantly lower CHIT1 concentration in heterozygotes compared to wild-type carriers, with conflicting reports in the literature [22–24]. However, this situation reflects clinical routine, where genotype information is usually not available. While genetic testing would be the best approach to ensure optimal efficacy of CHIT1 as a biomarker, the implementation of mandatory genetic testing would have a significant impact on the feasibility and availability of CHIT1 as a biomarker in routine diagnostic procedures. The results presented here suggest a satisfactory performance of CHIT1 as mitochondrial biomarker, even when heterozygous individuals are included. However, the expectation of elevated CHIT1 levels in mitochondriopathies may result in some heterozygous or homozygous mitochondriopathy patients not being identified. This may be seen as another argument for biomarker combinations, as discussed above.

In conclusion, serological assessment in a large cohort of patients with neuromuscular diseases suggests that CHIT1 levels are significantly elevated in mitochondriopathies and may complement the established biomarkers FGF21 and GDF15. The applicability of CHIT1 as a monitoring biomarker should be investigated in future longitudinal studies including larger cohorts.

## Supplementary Information

Below is the link to the electronic supplementary material.

Supplementary file1 (DOCX 76 KB)

## Data Availability

Anonymized data not published within this article will be made available by request from any qualified investigator.
