# Supplementary material for: Serum chitotriosidase-1 (CHIT1) as candidate biomarker for mitochondriopathies
Source: J Neurol. 2025 Feb 1;272(2):180. doi: 10.1007/s00415-025-12916-5 (PMC11787199; doi:10.1007/s00415-025-12916-5)
Supplement: Supplementary file 1 — Supplementary file1 (DOCX 76 KB) [file 415_2025_12916_MOESM1_ESM.docx]

**Electronic supplementary material**

# **Article name:** **Serum chitotriosidase-1 (CHIT1) as candidate biomarker for mitochondriopathies**

#

**Authors:** Laura Foerster, Leila Scholle, Tobias Mayer, Ilka Schneider, Gisela Stoltenburg-Didinger, Karl-Stefan Delank, Torsten Kraya, Andreas Hahn, David Strube, Anna Katharina Kölsch, Steffen Naegel, Lorenzo Barba, Alexander E. Volk, Markus Otto, Alexander Mensch

**Submitting and corresponding author:**

Alexander Mensch, MD

Department of Neurology, University Medicine Halle

Ernst-Grube-Str. 40, 06120 Halle (Saale), Germany

Tel.: +49 345 557 2856, Fax: +49 345 557 2860

E-Mail: [alexander.mensch@medizin.uni-halle.de](mailto:alexander.mensch@medizin.uni-halle.de)


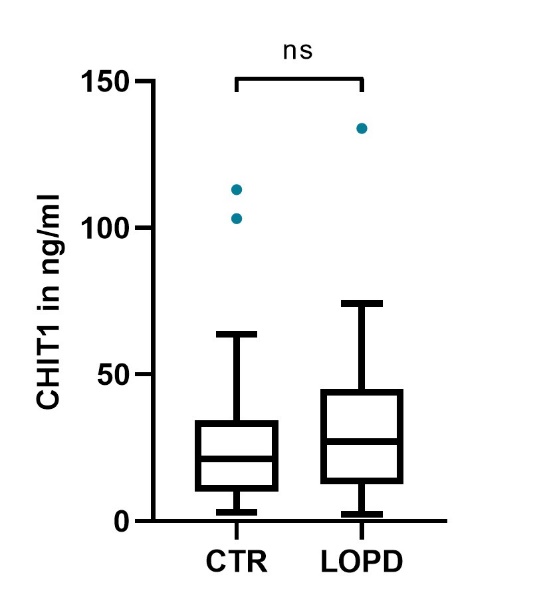


**Suppl. Fig. 1** CHIT1 concentration in LOPD vs CTR

CTR: healthy controls; LOPD: late-onset Pompe disease

**Suppl. Tab. 1** Clinical information mitochondriopathies

|  | no | age | sex | age at onset | disease duration | clinical phenotype | | genotype | | heteroplasmy |
| --- | --- | --- | --- | --- | --- | --- | --- | --- | --- | --- |
|  | 1 | 83 | female | n.a. | n.a. | ataxia | | point mutation (m.622G>A) | | n.d. |
|  | 2 | 64 | female | 14 | 50 | CPEO | | single large-scale mtDNA deletion (2 kb) | | n.d. |
|  | 3 | 65 | male | 28 | 37 | CPEO | | single large-scale mtDNA deletion (7 kb) | | 33% |
|  | 4 | 56 | female | 21 | 35 | CPEO | | single large-scale mtDNA deletion (4,5 kb) | | n.d. |
|  | 5 | 49 | female | 34 | 15 | CPEO | | single large-scale mtDNA deletion (6,3 kb) | | 70% |
|  | 6 | 56 | male | 12 | 44 | CPEO | | single large-scale mtDNA deletion (5 kb) | | 40% |
|  | 7 | 60 | male | 17 | 43 | CPEO | | single large-scale mtDNA deletion (7,25 kb) | | n.d. |
|  | 8 | 55 | male | 19 | 36 | CPEO | | single large-scale mtDNA deletion (4 kb) | | 40% |
|  | 9 | 75 | female | 17 | 58 | CPEO | | single large-scale mtDNA deletion (5 kb) | | n.d. |
|  | 10 | 65 | female | 17 | 48 | CPEO | | single large-scale mtDNA deletion (2 kb) | | n.d. |
|  | 11 | 70 | female | 15 | 55 | CPEO | | single large-scale mtDNA deletion (5,5 kb) | | n.d. |
|  | 12 | 43 | female | 13 | 30 | CPEO | | single large-scale mtDNA deletion (5 kb) | | n.d. |
|  | 13 | 27 | female | 13 | 14 | CPEO | | single large-scale mtDNA deletion (7,5 kb) | | n.d. |
|  | 14 | 63 | female | n.a. | n.a. | CPEO | | single large-scale mtDNA deletion (5 kb) | | n.d. |
|  | 15 | 70 | female | n.a. | n.a. | CPEO | | multiple large-scale mtDNA deletions; POLG1 pathogenic variant | | - |
|  | 16 | 65 | female | 3 | 62 | CPEO | | multiple large-scale mtDNA deletions; no pathogenic variant identified in nDNA | | - |
|  | 17 | 38 | female | 9 | 29 | CPEO | | multiple large-scale mtDNA deletions; no pathogenic variant identified in nDNA | | - |
|  | 18 | 81 | female | 9 | 72 | CPEO | | multiple large-scale mtDNA deletions; no pathogenic variant identified in nDNA | | - |
|  | 19 | 82 | female | 35 | 47 | CPEO-plus | | single large-scale mtDNA deletion (5 kb) | | n.d. |
|  | 20 | 55 | female | 12 | 43 | CPEO-plus | | single large-scale mtDNA deletion (2,5 kb) | | n.d. |
|  | 21 | 36 | female | 22 | 14 | CPEO-plus | | single large-scale mtDNA deletion (5 kb) | | 60% |
|  | 22 | 32 | female | 19 | 13 | CPEO-plus | | single large-scale mtDNA deletion (6,5 kB) | | 40% |
|  | 23 | 69 | female | 15 | 54 | CPEO-plus | | single large-scale mtDNA deletion (2,5 kb) | | n.d. |
|  | 24 | 70 | male | 24 | 46 | CPEO-plus (KSS) | | single large-scale mtDNA deletion (5 kb) | | 51% |
|  | no | age | sex | age at onset | disease duration | | clinical phenotype | | genotype | heteroplasmy |
|  | 25 | 26 | male | 20 | 6 | | LHON | | point mutation (m.3460G>A) | n.d. |
|  | 26 | 23 | female | 18 | 5 | | LHON | | point mutation (m.3460G>A) | n.d. |
|  | 27 | 45 | female | 34 | 11 | | LHON | | point mutation (m.14502T>C) | n.d. |
|  | 28 | 43 | female | n.a. | n.a. | | MELAS | | point mutation (m.3243A>G) | 20 |
|  | 29 | 19 | male | 13 | 6 | | MELAS | | point mutation (m.3243A>G) | 95 |
|  | 30 | 47 | female | n.a. | n.a. | | MELAS | | point mutation (m.3243A>G) | 12,5 |
|  | 31 | 18 | male | 6 | 12 | | MELAS | | point mutation (m.3243A>G) | 45 |
|  | 32 | 29 | female | 1 | 28 | | MELAS | | point mutation (m.3243A>G) | 77,5 |
|  | 33 | 66 | female | 44 | 22 | | MELAS | | point mutation (m.3243A>G) | 10 |
|  | 34 | 63 | male | 41 | 22 | | MELAS | | point mutation (m.3243A>G) | n.d. |

***CPEO:*** *chronic progressive external ophthalmoplegia;* ***LHON:*** *Leber's hereditary optic neuropathy;* ***KSS:*** *Kearns–Sayre syndrome;* ***MELAS:*** *Mitochondrial Encephalopathy, Lactic Acidosis, and Stroke-like episodes;* ***mtDNA:*** *mitochondrial DNA;* ***nDNA*** *- nuclear DNA;* ***n.a.:*** *not available;* ***n.d.:*** *not determined*

**Suppl. Tab. 2** Genetic information CHIT1 polymorphism

|  | subgroup |  |  | CHIT1-Polymorphism per subgroup* | | | | | |  | |  |
| --- | --- | --- | --- | --- | --- | --- | --- | --- | --- | --- | --- | --- |
|  | *number total* | *number tested* | | *wildtype* | | *heterozygous* | | *homozygous* | | | |  |
| hereditary myopathies | 90 | 34 | (38%) | 25 | (74%) | 9 | (26%) | | 0 | | (0%) | |
| inflammatory myopathies | 27 | 19 | (70%) | 12 | (63%) | 6 | (32%) | | 1 | | (5%) | |
| mitochondrial myopahies | 34 | 22 | (65%) | 16 | (73%) | 4 | (18%) | | 2 | | (9%) | |
| healthy controls | 38 | 6 | (16%) | 2 | (33%) | 3 | (50%) | | 1 | | (17%) | |

**percentages in brackets indicate the percentage of all tested specimen per subgroup*

**Suppl. Tab. 3** Linear regression analysis of CHIT1-influencing factors

|  | Variables | Serum chitotriosidase (ng/ml) | | |  |  |  |
| --- | --- | --- | --- | --- | --- | --- | --- |
|  |  | simple linear regression | |  | multiple linear regression | |  |
|  |  | ß | 95% CI | p-value | ß | 95% CI | p-value |
|  | CRP | 0.667 | 0.132 to 1.201 | 0.0148 | 0.607 | 0.098 to 1.116 | 0.0197 |
|  | Age | 0.650 | 0.360 to 0.941 | <0.0001 | 0.593 | 0.306 to 0.881 | <0.0001 |
|  | CTR vs. NMD | 17.07 | 4.754 to 29.38 | 0.0069 | 12.13 | 0.329 to 23.93 | 0.044 |

***CTR:*** *healthy controls;* ***NMD:*** *neuromuscular disorders including hereditary, inflammatory and mitochondrial pathologies;* ***CRP:*** *C-reactive protein;* ***95% - CI:*** *95% - confidence interval*

**Suppl. Tab. 4** CHIT1, FGF21 and GDF15 concentrations in neuromuscular diseases in comparison to healthy controls

|  | **CTR** | **HER** | **INF** | **MITO** |  |
| --- | --- | --- | --- | --- | --- |
| **CHIT1** (ng/ml) | **27.77** ± 24.62 | **37.11** ± 27.84 | **41.65** ± 25.77 | **68.32***** ± 48.42 |  |
|  |  |  |  |  |  |
|  | *n = 36* | *n = 87* | *n = 25* | *n = 32* |  |
| **FGF21** (pg/ml) | **211.90**  ± 188.80 | **300.20** ± 303.60 | **310.5** ± 306.20 | **493.50****** ± 268.10 |  |
|  |  |  |  |  |  |
|  | *n = 35* | *n = 44* | *n = 27* | *n = 26* |  |
| **GDF15** (pg/ml) | **844.40** ± 539.20 | **1013.00** ± 595.70 | **1584.00**** ± 905.70 | **2828.00****** ± 1075.00 |  |
|  |  |  |  |  |  |
|  | *n = 34* | *n = 34* | *n = 27* | *n =25* |  |

*p < 0.05*; p < 0.01**; p < 0.001***; p < 0.0001****.*

*Abbreviations:* ***CHIT1:*** *chitotriosidase 1;* ***FGF21:*** *fibroblast growth factor 21;* ***GDF15:*** *growth differentiation factor 15;* ***CTR:*** *healthy controls;* ***HER:*** *hereditary myopathies;* ***INF:*** *inflammatory myopathies;* ***MITO:*** *mitochondriopathies*

**Suppl. Tab. 5** CHIT1, FGF21 and GDF15 concentrations in different subgroups of mitochondriopathies

|  | **CTR** | **CPEO** | **CPEO+** | **LHON** | **MELAS** | **ataxia** |  |
| --- | --- | --- | --- | --- | --- | --- | --- |
| **CHIT1** (ng/ml) | **27.77** ± 24.62 | **82.51**** ± 56.39 | **68.87** ± 29.14 | **17.76** ± 14.45 | **56.59** ± 41.29 | **59.98** ± 0.00 |  |
|  |  |  |  |  |  |  |  |
|  | *n = 36* | *n = 16* | *n = 6* | *n = 3* | *n = 6* | *n = 1* |  |
| **FGF21** (pg/ml) | **199.6** ± 186.2 | **640.9****** ± 320.0 | **608.7**** ± 147.1 | **106.5** ± 92.63 | **278.5** ± 280.4 | **408.0** ± 0.00 |  |
|  |  |  |  |  |  |  |  |
|  | *n = 38* | *n = 17* | *n = 6* | *n = 2* | *n = 6* | *n = 1* |  |
| **GDF15** (pg/ml) | **815.1** ± 492.6 | **2692.0****** ± 1150.0 | **4002.0***** ± 1750.0 | **521.0** ± 1075.0 | **1813.0** ± 1563.0 | **2282.0** ± 0.00 |  |
|  |  |  |  |  |  |  |  |
|  | *n = 37* | *n = 17* | *n = 6* | *n = 2* | *n = 6* | *n = 1* |  |

*Level of significance is indicated as follows: p < 0.05*; p < 0.01**; p < 0.001***; p < 0.0001****.*

*Abbreviations:* ***CTR:*** *healthy controls;* ***CPEO:*** *chronic progressive external ophthalmoplegia;* ***LHON:*** *Leber hereditary optic neuropathy;* ***MELAS:*** *mitochondrial encephalomyopathy with lactic acidosis and stroke-like episodes*

**Suppl. Tab. 6** ROC analyses of CHIT1, FGF21, GDF15

| **Biomarker** | **Group** | **AUC** | **P-value** | **Cut-off** | **Sensitivity % (95% CI)** | **Specificity %  (95% CI)** |
| --- | --- | --- | --- | --- | --- | --- |
| **CHIT1**  (ng/ml) | CTR vs MITO | 0.7700 | 0.0001 | > 45.38 | **65.63** (48.31% - 79.59%) | **88.89** (74.69% - 95.59%) |
|  | HER vs MITO | 0.6810 | 0.0025 | > 44.53 | **65.63** (48.31% - 79.59%) | **70.11** (59.81% - 78.72%) |
|  | INF vs MITO | 0.6475 | 0.0577 | / | / | / |
|  | DC vs MITO | 0.6735 | 0.0028 | > 45.38 | **65.63** (48.31% - 79.59%) | **70.54** (61.53% - 78.18%) |
| **FGF21** (pg/ml) | CTR vs MITO | 0.8176 | < 0.0001 | > 343.50 | **73.08** (53.92% - 86.30%) | **88.57** (74.05% - 95.46%) |
|  | HER vs MITO | 0.7408 | 0.0008 | > 351.50 | **73.08** (53.92% - 86.30%) | **72.73** (58.15% - 83.65%) |
|  | INF vs MITO | 0.7457 | 0.0021 | > 294.50 | **76.92** (57.95% - 88.97%) | **70.37** (51.52% - 84.15%) |
|  | DC vs MITO | 0.7427 | 0.0003 | > 353.50 | **73.08** (53.92% - 86.30%) | **73.24** (61.95% - 82.15%) |
| **GDF15** (pg/ml) | CTR vs MITO | 0.9547 | < 0.0001 | > 1875.00 | **92.00** (75.03% - 98.58%) | **97.06** (85.08% - 99.85%) |
|  | HER vs MITO | 0.9224 | < 0.0001 | > 1816.00 | **92.00** (75.03% - 98.58%) | **91.18** (77.04% - 96.95%) |
|  | INF vs MITO | 0.8119 | 0.0001 | > 1798.00 | **92.00** (75.03% - 98.58%) | **70.37** (51.52% - 84.15%) |
|  | DC vs MITO | 0.8734 | < 0.0001 | > 1816.00 | **92.00** (75.03% - 98.58%) | **81.97** (70.53% - 89.62%) |

***AUC:*** *area under the curve;* ***95% CI:*** *95% confidence interval;* ***CHIT1:*** *chitotriosidase 1;* ***FGF21:*** *fibroblast growth factor 21;* ***GDF15:*** *growth differentiation factor 15;* ***CTR:*** *healthy controls;* ***HER:*** *hereditary myopathies;* ***INF:*** *inflammatory myopathies;* ***MITO:*** *mitochondriopathies*

**Suppl. Tab. 7** Diagnostic performance of several biomarker combinations

|  |  | CTR vs. MITO | | DC vs. MITO | |
| --- | --- | --- | --- | --- | --- |
|  |  | AUC | 95%-CI | AUC | 95%-CI |
|  | CHIT1 + FGF21 | 0.882 | 0.798 - 0.967 | 0.757 | 0.641 - 0.872 |
|  | CHIT1 + GDF15 | 0.965 | 0.925 - 0.999 | 0.914 | 0.851 - 0.977 |
|  | FGF21 + GDF15 | 0.957 | 0.905 - 0.999 | 0.887 | 0.816 - 0.959 |
|  | CHIT1 + FGF21 + GDF15 | 0.967 | 0.929 - 0.999 | 0.921 | 0.861 - 0.981 |

***CHIT1:*** *chitotriosidase 1;* ***FGF21:*** *fibroblast growth factor 21;* ***GDF15:*** *growth differentiation factor 15;* ***CTR:*** *healthy controls;* ***MITO:*** *mitochondriopathies;* ***DC:*** *diseased controls (including hereditary and inflammatory myopathies);* ***AUC:*** *area under the curve;* ***95% - CI:*** *95% - confidence interval*
